# MiRNA-363-3p/DUSP10/JNK axis mediates chemoresistance by enhancing DNA damage repair in diffuse large B-cell lymphoma

**DOI:** 10.1038/s41375-022-01565-6

**Published:** 2022-04-29

**Authors:** Wenping Zhou, Yuanlin Xu, Jiuyang Zhang, Peipei Zhang, Zhihua Yao, Zheng Yan, Haiying Wang, Junfeng Chu, Shuna Yao, Shuang Zhao, Shujun Yang, Yongjun Guo, Jinxin Miao, Kangdong Liu, Wing C. Chan, Qingxin Xia, Yanyan Liu

**Affiliations:** 1 Department of Internal Medicine, Affiliated Cancer Hospital of Zhengzhou University & Henan Cancer Hospital, Zhengzhou, Henan China; 2 Department of Lymphoma Research, Henan Cancer Institute, Zhengzhou, Henan China; 3 Department of Molecule and Pathology, Affiliated Cancer Hospital of Zhengzhou University & Henan Cancer Hospital, Zhengzhou, Henan China; 4 Academy of Chinese Medical Sciences, Henan University of Chinese Medicine, Zhengzhou, Henan China; 5 China-US (Henan) Hormel Cancer Institute, Zhengzhou, Henan China; 6 Department of Pathology, City of Hope National Medical Center, Duarte, CA USA

**Keywords:** Oncogenes, Genetics research

## Abstract

Anthracycline-based chemotherapy resistance represents a major challenge in diffuse large B-cell lymphoma (DLBCL). MiRNA and gene expression profiles (*n* = 47) were determined to uncover potential chemoresistance mechanisms and therapeutic approaches. An independent correlation between high expression of miRNA-363-3p and chemoresistance was observed and validated in a larger cohort (*n* = 106). MiRNA-363-3p was shown to reduce doxorubicin-induced apoptosis and tumor shrinkage in in vitro and in vivo experiments by ectopic expression and CRISPR/Cas9-mediated knockout in DLBCL cell lines. DNA methylation was found to participate in transcriptional regulation of miRNA-363-3p. Further investigation revealed that dual specificity phosphatase 10 (DUSP10) is a target of miRNA-363-3p and its suppression promotes the phosphorylation of c-Jun N-terminal kinase (JNK). The miRNA-363-3p/DUSP10/JNK axis was predominantly associated with negative regulation of homologous recombination (HR) and DNA repair pathways. Ectopic expression of miRNA-363-3p more effectively repaired doxorubicin-induced double-strand break (DSB) while enhancing non-homologous end joining repair and reducing HR repair. Targeting JNK and poly (ADP-ribose) polymerase 1 significantly inhibited doxorubicin-induced DSB repair, increased doxorubicin-induced cell apoptosis and tumor shrinkage, and improved the survival of tumor-bearing mice. In conclusion, the miRNA-363-3p/DUSP10/JNK axis is a novel chemoresistance mechanism in DLBCL that may be reversed by targeted therapy.

## Introduction

Diffuse large B-cell lymphoma (DLBCL) is the most common lymphoma with high heterogeneity in genetic abnormalities that lead to remarkable variations in clinical manifestation, therapy response, and outcome [1, 2]. Anthracycline-based chemotherapy (i.e., cyclophosphamide, vincristine, doxorubicin, and prednisone (CHOP)) is the mainstay for the treatment of DLBCL with long-term survival or cure in half of patients [3]. The addition of rituximab (R), a chimeric anti-CD20 monoclonal antibody, to CHOP (R-CHOP) chemotherapy further increases long-term survival by about 20% [4, 5]. Nevertheless, it unmet the clinical need of refractory and relapsed patients [6]. This study explored the resistance mechanisms and solutions of anthracycline-based chemotherapy resistance; resistance was defined as failure to achieve complete remission or relapse within six months after the end of treatment.

MicroRNAs (miRNAs) are endogenous, small, and non-coding RNA molecules (~22 nucleotides) that can repress the expression of a set of genes via binding to partially complementary sites in the 3′-untranslated region (UTR) of target transcripts. MiRNAs have been recognized to effectively distinguish different B-cell lymphomas, and to participate in the development, progression, and clinical response of the disease [7–11]. The miRNA-17-92 cluster is a paradigm, which is located on chromosome 13 and highly expressed in DLBCL with 13q31-q32 amplification [8, 12]. The cooperation of the miRNA-17-92 cluster with MYC can accelerate the genesis of B-cell lymphoma [8, 13]. Notably, the miRNA-106a-363 cluster, another member of miRNA-17-92 family located on chromosome X, has also been reported to exert a potential oncogenic role [14, 15]. It generates 6 mature miRNAs (miRNA-106a, miRNA-18b, miRNA-19b-2, miRNA-20b, miRNA-92a-2, and miRNA-363-3p), and is speculated to have unique and overlapping functions with the miRNA-17-92 cluster [16]. Our previous study revealed a significant association of miRNA-363-3p with R-CHOP therapy failure in DLBCL [17]. Further studies showed that miRNA-363-3p-mediated suppression of its target gene calcium channel alpha1 C (CACNA1C) plays an important role in rituximab resistance, by reducing rituximab-mediated calcium influx and DLBCL cell apoptosis [18]. Herein, we extended our study of miRNA-363-3p and found that it contributed to the resistance of DLBCL to anthracycline-based chemotherapy. A relevant resistance mechanism involving miRNA-363-3p/dual-specificity phosphatase 10 (DUSP10)/c-Jun N-terminal kinase (JNK)-mediated DNA damage repair was identified. This resistance mechanism was independent of cell-of-origin (COO) and previously known aberrations in MYC, BCL2, and TP53 [19–24]. Moreover, miRNA-363-3p was found to be transcriptionally modulated by DNA methylation. Therefore, we propose the development of novel approaches to overcome the chemoresistance by reducing miRNA-363-3p expression or inhibiting JNK and poly (ADP-ribose) polymerase 1 (PARP1) activity.

## Materials and methods

### Patient samples and cell culture

Cryopreserved tissues from 47 DLBCL cases were profiled for gene expression and miRNA expression [17]. In addition, 106 patients with adequate formalin-fixed and paraffin-embedded samples were enrolled and their clinicopathological and molecular features are described in Supplementary Table 1. All of patients received rituximab plus anthracycline-based chemotherapy. The diagnosis of DLBCL was confirmed by at least two pathologists according to the World Health Organization classification [25]. Written informed consent was obtained from all patients, and this study was reviewed and approved by the Ethics Committee of Affiliated Cancer Hospital of Zhengzhou University & Henan Cancer Hospital (License number 2019341), and was conducted in accordance with the Declaration of Helsinki.

Human DLBCL cell lines OCI-Ly8, OCI-Ly3, OCI-Ly7, DOHH2, Val, and Ros50 were cultured in IMDM (Gibco). They have been authenticated and regularly monitored for mycoplasma contamination. Single-cell suspensions of tumor samples from three DLBCL patients were prepared according to the reference protocol [26]. Normal primary B cells were isolated from fresh tonsils of male patients as described in previous studies [17]. Briefly, B-cell subsets were isolated by CD19 MicroBeads (Invitrogen), and naive (IgD^+^ CD38^lo^ CD27^−^), centroblast (CD77^+^ CD38^hi^), and centrocyte (CD77^–^ CD38^hi^) B cells were obtained using fluorescence-activated cell sorting.

### MiRNA and gene expression profiling (GEP)

Related methods have been described in previous studies [17, 23, 27]. Briefly, total RNA for miRNA expression was extracted using miRNeasy Mini kit (Qiagen). Reverse-transcription was performed using Megaplex RT Primer (Life Technologies), and miRNA expression was quantitated using TaqMan human microRNA array V2.0 (Applied Biosystems). Total RNA for GEP was extracted using Allprep DNA/RNA extraction kit (Qiagen) and analyzed using GeneChip Human Genome U133 Plus 2.0 Array (Affymetrix).

### Quantitative real-time (RT) PCR

Total RNA was isolated using miRNeasy Mini kit (Qiagen) or MagMAX FFPE DNA/RNA Ultra kit (Applied Biosystems) and reversely transcribed by miScript II RT kit (Qiagen). RT PCR was performed using miScript SYBR Green PCR kit (Qiagen) with specific primers (Supplementary Table 2).

### Immunohistochemistry

Immunohistochemical staining was performed to detect the expression of CD10 (Abcam; 1:500), BCL6 (Abcam; 1:250), MUM1 (Abcam; 1:250), TP53 (Abcam; 1:500), MYC (Santa Cruz; 1:200), BCL2 (Abcam; 1:500), and DUSP10 (Abcam; 1:500) in the DLBCL cohort (*n* = 106). This method has been described in our previous study [28], and primary and secondary antibodies used are listed in Supplementary Table 3.

### Plasmids and cell transduction

The DNA sequence containing miRNA-363-3p (human GRCh38/hg38 version, ChrX:134,169,177-134,169,688; 512 bp) was cloned from the human genome and inserted into the lentiviral vector FUA-EGFP from pCDH-EF1-MCS (System Bioscience) by digesting BamHI/EcoRI under the control of the Ubc promoter (Supplementary Fig. 1A). Lentiviruses were packaged in 293 T cells with pSPAX2 and pMD2G using Lipofectamine 2000 (Invitrogen) and collected by ultracentrifugation. Puromycin (2 µg/ml) was used to select infected cells. The pSpCas9 (BB)−2A-GFP (PX458) was obtained from Addgene and inserted into the multiple clone site to generate PX458M. Four individual sgRNAs were designed near the 5′ and 3′ ends of the palindrome arm. The PX458M containing two sgRNAs was transduced into cells using the Lonza Nucleofector system (Supplementary Fig. 1B). EGFP-positive cells were sorted by flow cytometry and then diluted into 96-well plates. Single clones were validated by PCR and further used for sequencing.

### Validation of promoter

The promoter sequence of candidate miRNA-363-3p (ChrX:134,171,605-134,172,956; 1,352 bp) was amplified from the human genome using specific primers (Supplementary Table 2) and cloned into the pGL3-Enhancer vector (Promega) by restriction digestion using KpnI/BglII. Insert orientation was verified by sequencing with RVprimer3 primers. The pGL3-Enhancer vector lacking a promoter sequence (Promega) and the pGL3-Control vector containing SV40 promoter (Promega) were constructed as negative and positive controls, respectively (Supplementary Fig. 2A–C). They were cotransfected with renilla luciferase vector into 293 T cells by Lipofectamine 2000 (Invitrogen). Luciferase assays were performed using the Dual Luciferase Reporter Assay System (Promega).

### Chromatin immunoprecipitation

Intracellular protein-DNA complexes were cross-linked in situ with 1% formaldehyde. Total lysates were sonicated by Bioruptor Pico at 45 s on/off for 15 min and subjected to chromatin-conjugated immunoprecipitation using an antibody specific for human MYC (Santa Cruz; 200 μg/ml) (Supplementary Table 3). After reversal of cross-links, the precipitated DNA was purified and analyzed for miRNA-363-3p by quantitative PCRwith specific primers (Supplementary Table 2).

### Bisulfite sequencing PCR

Genomic DNA was extracted using the procedure described previously [29]. One microgram of DNA solution was treated with the EZ DNA Methylation-Gold kit (Zymo Research) according to the manufacturer’s instruction. Two sets of miRNA-363-3p primers were designed to amply target regions using touch-down PCR (Supplementary Table 2). The amplified product (330 bp) was purified and cloned into the PMD19-T vector (Takara). Five clones were sequenced with M13 primers to analyze CpG islands (ChrX: 134,172,059 − 134,172,163; 105 bp).

### Validation of miRNA gene targets

The sequence of mature human miRNA-363-3p (Accession no: MI0000764) was retrieved from the miRNA database (http://www.mirbase.org). Target transcripts for miRNA-363-3p were predicted using the TargetScan 7.0 Human database and collected using default setting [30–32]. The validation was performed by cotransfecting pmirGLO Dual-Luciferase miRNA target expression vector (pmirGLO) (Promega) containing wild-type or mutant 3′UTR (annealed synthetic oligonucleotides) (Supplementary Table 2) with miRNA-363-3p mimic (5′-AAUUGCAGGUAUCCAUCUGUA-3′; 20 nM) into 293 T cells using Lipofectamine 2000 (Invitrogen). Luciferase activity was analyzed in triplicate per group.

### Western blot

Total protein was extracted by lysing cells in NP40 buffer containing 1× protease inhibitor cocktail (Roche) and phosphatase inhibitor cocktail 2 (Sigma). Nuclear fractions were prepared using hypotonic buffer according to a previously described protocol [33]. Immunoblotting was carried out with a standard protocol with primary antibodies including histone H3 (Abcam; 1:2,000), DUSP10 (Abcam; 1:1,000), γH2AX (Ser139; Abcam; 1:2,000), JNK (CST; 1:1,000), phospho-JNK (CST; 1:1,000), p38 (CST; 1:1,000), phospho-p38 (CST; 1:1,000), p53 (Abcam; 1:1,000), phospho-p53 (Abcam; 1:1,000), Bcl2 (Abcam; 1:2,000), ATF2 (Santa Cruz; 1:1,000), phospho-ATF2 (Santa Cruz; 1:1,000), and MYC (Santa Cruz; 1:1,000) (Supplementary Table 3). The protein bands were visualized using the enhanced chemiluminescence system (Millipore) and imaged with the Odyssey Infrared Imaging System (Li-Cor Biosciences).

### Immunofluorescence

After drug treatment, cells were fixed in 4% paraformaldehyde and then permeabilized by 0.1% Triton X-100 for 15 min. Immunofluorescence was performed with anti-human γH2AX (Ser139) primary antibody (CST; 1:400) and then with Alexa Fluor 488-conjugated secondary antibody (Invitrogen; 1:500) (Supplementary Table 3). Hoechst 33342 was used for nuclear staining (blue). Fluorescence images were collected using a ZEISS LSM 780 confocal microscope (Zeiss).

### Cell apoptosis

Cell apoptosis was analyzed using PE Annexin V Apoptosis Detection Kit I (BD Bioscience) according to the manufacturer’s instruction. Fluorescent signals were collected on a BD FACSCalibur (BD Bioscience) and data were analyzed with FlowJo v.10 Software. Drug synergy was determined by the combination index (CI = (D) 1/(D_X_) 1 + (D) 2/(D_X_) 2) with the Chou-Talalay method using CompuSyn software [34]. CI < 1, = 1, and > 1 indicate synergistic, additive, and antagonistic effects, respectively.

### HR, NHEJ, and a-NHEJ repair assay

HR, NHEJ, and a-NHEJ assays were performed in 293 T cells as described previously [35]. Briefly, 1 × 10^5^ cells stably expressing Active/Repressed TLR (AR-TLR) or single-strand annealing TLR (SSA-TLR) reporter were seeded in a 24-well plate for 24 h prior to transfection. I-SceI nuclease expression plasmid was transiently co-transfected with miRNA-363-3p mimic (5′-AAUUGCAGGUAUCCAUCUGUA-3′; 20 nM) or antisense inhibitor (5′-UACAGAUGGAUACCGUGCAAUU-3′; 20 nM). The ratio of EGFP and mCherry fluorescence was measured by flow cytometry to determine the efficiency of HR to NHEJ and HR to a-NHEJ.

### Xenograft mouse model

DLBCL cells were injected subcutaneously into one flank of six-week-old SCID mice (Charles River). When the tumor volume reached approximately 150 mm^3^, mice (*n* = 82) were randomized into control and experimental groups. Saline or doxorubicin (20 mg/kg) was administered intraperitoneally twice a week, SP600125 (Selleck; 25 mg/kg) injected intraperitoneally every other day, and BGB-290 (Biogene; 25 mg/kg) given orally once every two days. Animals were maintained and manipulated in accordance with the principles of laboratory animal care under the Institutional Animal Care and Use Committee-approved protocol. The study was reviewed and approved by the Ethics Committee on Animal Experiment of Henan University of Chinese Medicine (License DWLL20190501 and its amendments).

### Statistical analysis

Continuous and categorical variables were compared using *t*-test and *χ*^2^- test, respectively. Multivariate analysis was performed using logistic regression method. Progression-free survival (PFS) was calculated from the date of initial diagnosis to the time of recurrence, death or the last follow-up. Overall survival (OS) was measured from the date of initial diagnosis to the death or the last follow-up. PFS and OS were estimated by the Kaplan–Meier method and the log-rank test was used for comparison between groups. Statistical analysis was carried out using Statistical Package for the Social Sciences 21.0 software (SPSS). Statistical significance was defined as *p* < 0.05.

## Results

### High levels of miRNA-363-3p significantly correlate with anthracycline-based chemotherapy resistance

**Fig. 1:**
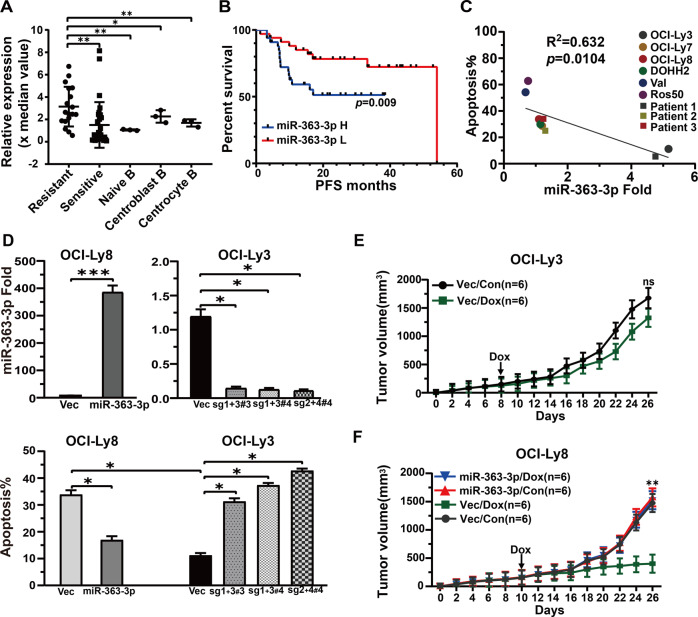
The significant association between high expression of miRNA-363-3p and chemoresistance. **A** The levels of miRNA-363-3p were higher in patients with R-CHOP resistance (*n* = 18) than in those with R-CHOP sensitivity (*n* = 29, **p < 0.01), and in each of naive (*n* = 3, **p < 0.01), centroblast (*n* = 3, *p < 0.05), and centrocyte (*n* = 3, *p < 0.05) B cells. **B** Kaplan–Meier and Log-Rank analyses showed the significant association of high miRNA-363-3p expression with unfavorable PFS (*p* = 0.009) in the larger DLBCL cohort (*n* = 106). **C** Quantitative RT PCR analysis showed an inverse correlation between high expression of miRNA-363-3p and doxorubicin-induced apoptosis in OCI-Ly3, OCI-Ly8, OCI-Ly7, DOHH2, Val, and Ros50 DLBCL cell lines and tumor samples from three DLBCL patients (*R*^2^ = 0.632, *p < 0.05). **D** Quantitative RT PCR analysis showed a significant increase in miRNA-363-3p expression in miRNA-363-3p-ectopic OCI-Ly8 cells (***p < 0.001) and a significant reduction in miRNA-363-3p-knockout OCI-Ly3 cells (*p < 0.05). The proportion of doxorubicin-induced apoptosis was significantly reduced in miRNA-363-3p-ectopic OCI-Ly8 cells (*p < 0.05) and increased in miRNA-363-3p-knockout OCI-Ly3 cells (*p < 0.05). **E** Doxorubicin induced a modest tumor suppression in OCI-Ly3 vector-bearing mice (*n* = 6 each). **F** Doxorubicin induced significant tumor suppression in OCI-Ly8 vector control-bearing mice (*n* = 6 each), which was significantly decreased in miRNA-363-3p-ectopic mice (*n* = 6 each) (**p < 0.01).The “#” symbol represents the single clone number of miRNA-363-3p-knockout cells.

MiRNA expression profiling was performed on cryopreserved tumor samples from 47 DLBCL patients. The level of miRNA-363-3p was significantly increased in the resistant group (*n* = 18) (*p* = 0.003), and also higher than that in the naive (*n* = 3, *p* < 0.01), centroblast (*n* = 3, *p* < 0.05), and centrocyte (*n* = 3, *p* < 0.05) B cells (Fig. 1A). Multivariate analysis including GEP-based COO classification and clinical parameters of IPI score showed an independent association of miRNA-363-3p with resistance. We did not find significant differences regarding other paralogs of miRNA-363-3p, which exhibited obvious differences in the secondary structure of precursor miRNA moieties (Supplementary Fig. 3A). The result was further confirmed in another DLBCL cohort (*n* = 106) (Supplementary Fig. 3B). Furthermore, patients with high miRNA-363-3p level displayed unfavorable PFS (*p* = 0.009) (Fig. 1B).

High variance in miRNA-363-3p levels was also observed in B-cell lymphoma cell lines. The level of miRNA-363-3p was negatively related to doxorubicin (25 ng/ml)-induced apoptosis in six DLBCL cell lines and three DLBCL patient tumor cells after 48 h of treatment (Fig. 1C). A significant decrease in doxorubicin-induced apoptosis was observed in miRNA-363-3p-ectopic OCI-Ly8 (*p* = 0.013), while a significant increase in apoptosis was detected in CRISPR/Cas9-mediated miRNA-363-3p-knockout OCI-Ly3 (*p* = 0.006) (Fig. 1D; Supplementary Figs. 3C, D, and 4). The relationship between miRNA-363-3p expression and doxorubicin resistance was confirmed by in vivo experiments. MiRNA-363-3p-knockout OCI-Ly3 was observed to have a low tumorigenesis efficiency (2/25, 8%) (Supplementary Fig. 5A) and therefore did not participate in the grouping. Indeed, cell viability assay showed that knockout of miRNA-363-3p significantly inhibited cell proliferation (Supplementary Fig. 5B). Tumor-bearing mice were divided into control and experimental groups (*n* = 6 each). Doxorubicin induced significant tumor shrinkage in mice bearing OCI-Ly8 vector compared with those bearing OCI-Ly3 vector (*p* < 0.01), and the efficacy of doxorubicin on OCI-Ly8 was significantly reduced by ectopic expression of miRNA-363-3p (Fig. 1E, F; Supplementary Fig. 5C, D).

### MiRNA-363-3p is transcriptionally modulated by DNA methylation other than MYC

**Fig. 2:**
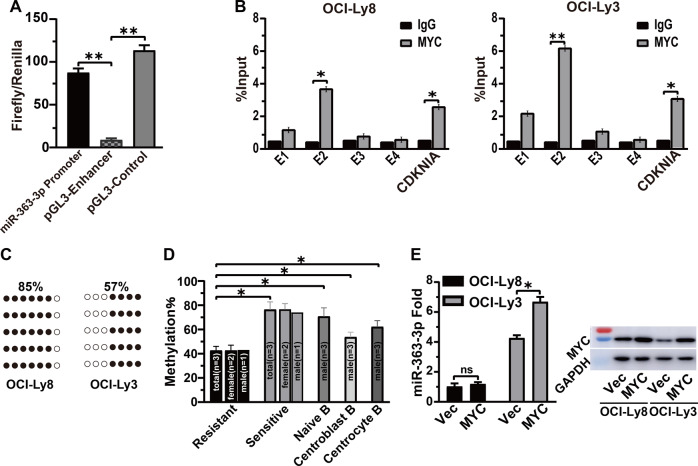
The transcriptional regulation of miRNA-363-3p. **A** Dual luciferase report assay showed that the firefly signals of pGL3-miRNA-363-3p promoter vector were higher than those of pGL3-enhancer vector (**p < 0.01). **B** Chromatin immunoprecipitation (ChIP) combining with PCR assay revealed precise binding of MYC to E-box-2 site and higher binding efficiency in OCI-Ly3 than in OCI-Ly8 cells. **C** Bisulfite Sequencing PCR (BSP) assay showed that CpG sites surrounding E-box-2 had higher levels of methylation in OCI-Ly3 than in OCI-Ly8 (●: Methylation, ○: Unmethylation). Each row represents a single cloned allele. **D** The analysis of CpG methylation showed that resistant patients (*n* = 3) had lower methylation than naive (*n* = 3), centroblast (*n* = 3), and centrocyte (*n* = 3) B cells and sensitive patients (*n* = 3) (*p < 0.05), respectively. Methylation levels in female and male patients are illustrated separately. **E** Quantitative RT PCR analysis found insignificant increase of miRNA-363-3p levels after ectopic expression of MYC in OCI-Ly3 cells.

A 1.258 kb sequence around the CpG island was predicted to be a candidate miRNA-363-3p promoter (Supplementary Fig. 6A), and its function was validated by significantly enhancing firefly luciferase activity compared to the negative control (Fig. 2A). Bioinformatics analysis revealed four E-box elements for MYC binding in the CpG island (Supplementary Fig. 6B). The E-box-2 was determined as the precise binding sites for MYC by chromatin immunoprecipitation plus PCR assay (Supplementary Fig. 6C). Notably, the binding level of MYC was higher in OCI-Ly3 than in OCI-Ly8 (Fig. 2B). Locus-specific DNA methylation analysis by bisulfite sequencing PCR uncovered a lower CpG methylation surrounding the E-box-2 in OCI-Ly3 than in OCI-Ly8 (57% vs 85%, *p* < 0.05) (Fig. 2C). After 6 days of treatment with the DNA methyltransferase inhibitor decitabine (0.1 μM), the methylation in OCI-Ly8 cells was gradually reduced, and the level of miRNA-363-3p was significantly increased (Supplementary Fig. 6D). The effect of methylation on the expression of miRNA-363-3p was further validated by observing that resistant patients (*n* = 3) had lower methylation levels than sensitive patients (*n* = 3) and naive (*n* = 3), centroblast (*n* = 3), and centrocyte (*n* = 3) B cells (Fig. 2D). The female to male ratio was 2:1 in both resistant and sensitive patients, balancing the effect of sex on DNA methylation of chromosome X. Moreover, the ectopic expression of MYC did not significantly increase the level of miRNA-363-3p in OCI-Ly8 compared with OCI-Ly3 (Fig. 2E). In summary, these results confirmed that miRNA-363-3p is transcriptionally regulated by DNA methylation other than MYC.

### MiRNA-363-3p directly inhibits DUSP10 expression and then enhances JNK phosphorylation

**Fig. 3:**
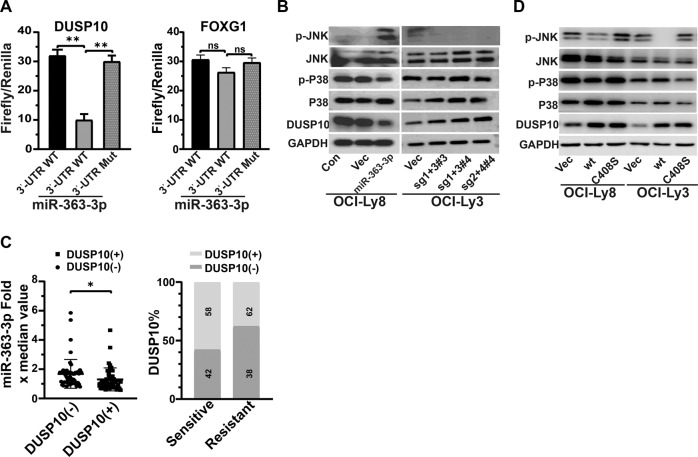
The role of DUSP10 in miRNA-363-3p-mediated chemoresistance. **A** Dual luciferase reporter assay showed that DUSP10 (**p < 0.01) is the target gene of miRNA-363-3p, but not FOXG1. **B** Western blot analysis verified that ectopic expression of miRNA-363-3p obviously decreased the levels of DUSP10 and increased the levels of phosphorylated JNK (p-JNK) in OCI-Ly8 cells, and the knockout of miRNA-363-3p displayed the opposite effect in OCI-Ly3 cells. **C** In the larger DLBCL cohort (*n* = 106), quantitative RT PCR analysis showed that the levels of miRNA-363-3p were higher in patients with negative DUSP10 protein (*n* = 52) than those with positive one (*n* = 54, *p < 0.01), and the negative DUSP10 protein was correlated with chemoresistance (*p* < 0.05). **D** Western blot assay showed that it was ectopic expression of wild-type (WT) DUSP10, not inactive DUSP10 (C408S), to increase the level of p-JNK. The “#” symbol represents the single clone number of miRNA-363-3p-knockout cells.

Unsupervised hierarchical clustering of GEP data from cryopreserved samples of 47 DLBCL patients divided the patients into two distinct subgroups (Supplementary Fig. 7A). All patients in one group (*n* = 16) were resistant, while most cases in the other group (29/31) were sensitive. A total of 78 upregulated (fold change > 2 and *p* < 0.01) and 135 downregulated genes (fold change < 0.5 and *p* < 0.01) were enriched to correlate with resistance. In addition, 278 potential target transcripts for miRNA-363-3p were predicted. When we overlapped downregulated genes with candidate target genes of miRNA-363-3p, *DUSP10*, *FOXG1*, and *CACNA1C* were identified (Supplementary Fig. 7B). To test the direct regulation of these genes by miRNA-363-3p, we cloned the 3′UTR of candidate genes with putative or mutant binding sites into a luciferase reporter plasmid. The result showed that the miRNA-363-3p mimic significantly repressed the luciferase activity of plasmids harboring the 3′UTR of *DUSP10* and *CACNA1C* genes (data published [18]), but not the 3′UTR of *FOXG1* gene (*n* = 3) (Fig. 3A). CACNA1C has been reported to affect rituximab resistance [18]. Here, we focused on the role of DUSP10 in chemoresistance. The prediction showed high total context ++ score (−0.55) and aggregate P_CT_ (0.95) for the miRNA-363-3p binding site within the 3′UTR of *DUSP10* **(**Supplementary Fig. 7C**)**. Western blot analysis confirmed that DUSP10 protein was decreased in miRNA-363-3p-ectopic OCI-Ly8 and increased in miRNA-363-3p-knockout OCI-ly3 (Fig. 3B). A negative correlation of DUSP10 expression with miRNA-363-3p (*p* = 0.032) and chemoresistance (*p* = 0.038) was validated in 106 DLBCL patients (Fig. 3C; Supplementary Fig. 7D). In another larger DLBCL cohort (*n* = 221) [36], patients with low *DUSP10* gene expression also displayed unfavorable PFS (*p* = 0.0005) and OS (*p* = 0.0001) (Supplementary Fig. 7E). These data confirmed that DUSP10 is a target gene of miRNA-363-3p and contributes to miRNA-363-3p-related resistance in DLBCL. JNK and p38 are negatively modulated by DUSP10-mediated dephosphorylation involving mitogen-activated protein kinases (MAPK) signaling pathways [37]. We found that the levels of phosphorylated JNK, but not phosphorylated p38, were enhanced in miRNA-363-3p-ectopic OCI-Ly8 and decreased in miRNA-363-3p-knockout OCI-Ly3 (Fig. 3B). The phosphatase-dead DUSP10 (C408S) further verified the function of miRNA-363-3p/DUSP10/JNK axis in DLBCL cells (Fig. 3D).

### MiRNA-363-3p-related chemoresistance is associated with enhanced doxorubicin-induced DNA double-strand break (DSB) repair

**Fig. 4:**
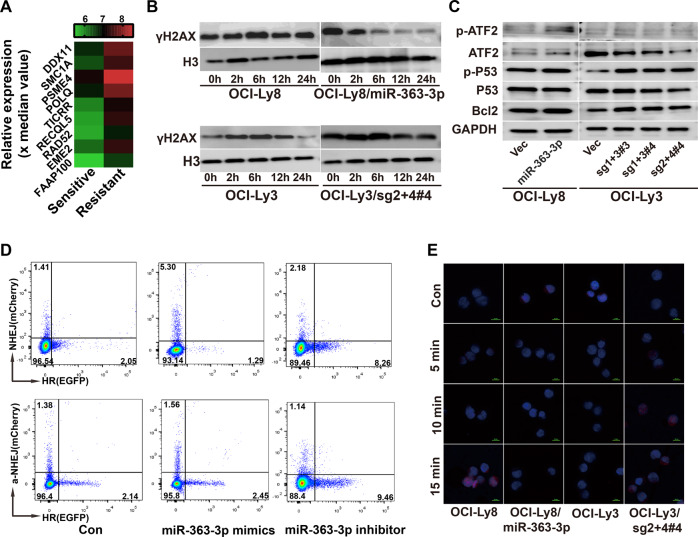
Aberrant DNA damage repair in miRNA-363-3p/DUSP10/JNK axis-related chemoresistance. **A** The heat map showed that 9 genes enriched in DNA repair pathway were upregulated in resistant patients. **B** Western blot assay showed gradual decrease in doxorubicin (25 ng/ml)-induced γH2AX levels in miRNA-363-3p-ectopic OCI-Ly8 cells and increase in miRNA-363-3p-knockout OCI-Ly3 cells after doxorubicin withdrawal of 0, 2, 6, 12, and 24 h. Histone 3 (H3) was used as internal control. **C** Western blot assay showed that the change of miRNA-363-3p did not alter the levels of TP53, phosphorylated TP53 and BCL2 proteins in miRNA-363-3p-ectopic OCI-Ly8 and miRNA-363-3p-knockout OCI-Ly3 cells. However, the levels of phosphorylated ATF2 was affected. **D** The miRNA-363-3p mimic decreased the HR and increased the NHEJ, alternative NHEJ (a-NHEJ) in 293 T cells, and its inhibitors showed opposite effects. **E** Immunofluorescence assays showed doxorubicin-induced RAD51 foci was significantly decreased in miRNA-363-3p-ectopic OCI-Ly8 cells and increased in miRNA-363-3p-knockout OCI-Ly3 cells after doxorubicin withdrawal for 0, 5, 10, and 15 min. The “#” symbol represents the single clone number of miRNA-363-3p-knockout cells.

David pathway analysis of 78 resistance-related upregulated (fold change > 2 and *p* < 0.01) genes revealed the top 10 pathways; DNA damage repair pathways were predominantly enriched, including the negative regulation of HR (*RECQL5*, *POLQ*; *p* = 0.0056) and DNA repair (*FAAP100*, *RECQL5*, *DDX11*, *TICRR*, *PSME4*, *POLQ*, and *SMC1A*; *p* = 0.0118) (Fig. 4A; Supplementary Fig. 8A), which were further validated in miRNA-363-3p-ectopic OCI-Ly8 and miRNA-363-3p-knockout OCI-Ly3 cells (Supplementary Fig. 8B). γH2AX is a known consensus biomarker for DSB. Doxorubicin-induced γH2AX levels in miRNA-363-3p-ectopic OCI-Ly8 cells gradually reduced at 0, 2, 6, 12, and 24 h after doxorubicin (25 ng/ml) treatment for 2 h, but not in OCI-Ly8 control cells. Conversely, miRNA-363-3p knockout resulted in increased doxorubicin-induced γH2AX levels in OCI-Ly3 cells (Fig. 4B). Changes in γH2AX levels were also observed by immunofluorescence assay (Supplementary Fig. 8C). However, we did not find the gene enrichment of *TP53* and apoptosis-related pathways in these patients with high miRNA-363-3p expression. The change of miRNA-363-3p expression did not influence the levels of TP53 and BCL2 proteins. As expected, the levels of ATF2 and phosphorylated ATF2 were positively related to miRNA-363-3p expression (Fig. 4C), a consensus target of JNK.

DSB is mainly repaired by HR and NHEJ, occasionally by the error-prone alternative NHEJ. Using a reporter plasmid system, we verified that the miRNA-363-3p mimic significantly decreased HR activity, but increased NHEJ and alternative NHEJ activity in 293 T cells; the miRNA-363-3p inhibitor showed opposite effects (Fig. 4D). RAD51 is a core molecule for HR repair. Immunofluorescence assay found that doxorubicin-induced RAD51 foci were obviously decreased by ectopic expression of miRNA-363-3p, but increased by knockout of miRNA-363-3p (Fig. 4E). MiRNA-363-3p-induced HR suppression was eventually confirmed.

### MiRNA-363-3p-related chemoresistance is reversed by inhibition of JNK and PARP1

**Fig. 5:**
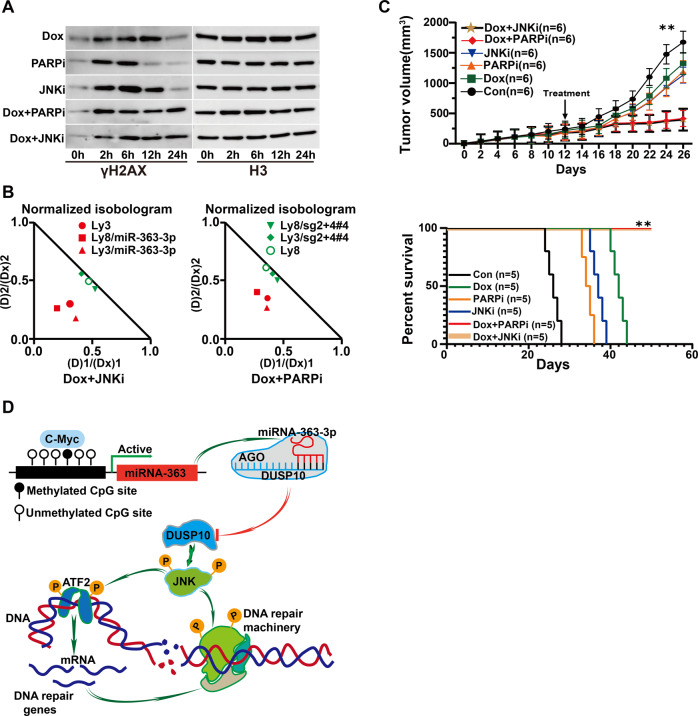
The effect of JNK and PARP1 inhibitors on doxorubicin resistance. **A** Western blot assay showed that SP600125 (20 µM, JNK1/2/3 inhibitor) and BGB-290 (25 µM, PARP1 inhibitor) significantly increased doxorubicin (25 ng/ml)-induced γH2AX levels in OCI-Ly3 cells after treatment for 0, 2, 6, 12, and 24 h. Histone 3 (H3) was used as internal control. **B** A significant synergy between doxorubicin and SP600125 (JNK1/2/3 inhibitor), BGB-290 (PARP1 inhibitor) was confirmed by the combination index (CI = (D) 1/(DX) 1 + (D) 2/(DX) 2) with the Chou-Talalay method using CompuSyn software. Synergistic, additive, and antagonistic effects are defined as CI < 1, CI = 1, and CI > 1, respectively. **C** SP600125 (25 mg/kg, JNK1/2/3 inhibitor) and BGB-290 (25 mg/kg, PARP1 inhibitor) significantly enhanced doxorubicin (20 mg/kg)-induced tumor suppression in mice bearing OCI-Ly3 cells (*n* = 6 each) (**p < 0.01). The addition of SP600125 (25 mg/kg, JNK1/2/3 inhibitor) and BGB-290 (25 mg/kg, PARP1 inhibitor) significantly improved the survival of mice compared with doxorubicin (20 mg/kg) alone (*n* = 5 each). **D** The schematic mechanism of miR-363-3p/DUSP10/JNK axis was shown. The “#” symbol represents the single clone number of miRNA-363-3p-knockout cells.

PARP1 inhibitors have been shown to have notable efficacy in HR-deficient cancer cells. Thus, the pan-JNK inhibitor SP600125 (20 µM) and the PARP1 inhibitor BGB-290 (25 µM) were evaluated for their potential to overcome chemoresistance associated with miRNA-363-3p/DUSP10/JNK axis in DLBCL cells. The result showed that they obviously enhanced doxorubicin (25 ng/ml)-induced γH2AX level in high miRNA-363-3p-expressed OCI-Ly3 cells using Western blot and immunofluorescence assays (Fig. 5A and Supplementary Fig. 9A). Consistently, flow cytometry analysis revealed that SP600125 and BGB-290 significantly promoted doxorubicin-induced apoptosis (Supplementary Fig. 9B). Furthermore, an obvious synergy between doxorubicin and SP600125 or BGB-290 was observed in high miRNA-363-3p-expressed cell lines, but not in those with low expression (Fig. 5B). Their efficacy was further confirmed in xenograft mouse models bearing OCI-Ly3 cells. The addition of SP600125 or BGB-290 significantly increased doxorubicin-induced tumor regression and survival (Fig. 5C; Supplementary Fig. 9C). In sum, we demonstrated that miRNA-363-3p-related chemoresistance can be reversed by inhibiting JNK and PARP1 in DLBCL cells. The intrinsic mechanism is schemed in Fig. 5D.

## Discussion

This study identified a unique association of miRNA-363-3p with chemoresistance in contrast to its paralogs from the miRNA-106a-363 cluster. A specific transcription regulation of miRNA-363-3p by DNA methylation other than MYC was confirmed, which was different from the miRNA-17-92 cluster and miRNA-18b, miRNA-20b, and miRNA-106a from the miRNA-106a-363 cluster that are upregulated by MYC [17]. The differential expression among miRNAs in the miRNA-106a-363 cluster has also been reported in another study [14]. It may be attributed to the distinct secondary structures of precursor miRNA moieties in the cluster [38, 39], indicating the complexity of miRNA cluster regulation and biogenesis. Furthermore, the dysregulation of miRNA-363-3p has been found to be associated with tumorigenesis, proliferation, and drug resistance in some solid tumors [40, 41]. The knockout of miRNA-363-3p was demonstrated to perform anti-tumor activity in the study. The development of miRNA delivery systems will push forward the clinical translation of miRNA therapy.

Increasing evidence suggests that miRNA-363-3p functions by targeting different signaling pathways in distinct cellular contexts [42, 43]. DUSP10, known as MAPK phosphatase (MKP) 5 [44], is a negative regulator of p38 and JNK [45]. In this study, we identified and validated that DUSP10 is a target of miRNA-363-3p and participates in miRNA-363-3p-related chemoresistance by regulating JNK phosphorylation in DLBCL cells. Activation of JNK can phosphorylate numerous proteins residing in mitochondria and nucleus, thereby regulating cell growth, differentiation, survival, and apoptosis [46–51]. We found that miRNA-363-3p-related JNK activation promoted chemotherapeutic drug-induced DSB repair by affecting its substrate ATF2. Chemotherapeutic drugs have been demonstrated to activate JNK and induce the transcription of many DNA repair genes by phosphorylating its substrates ATF2 and c-Jun [52, 53]. Moreover, JNK can phosphorylate SIRT6 to facilitate the recruitment of PARP1 at the DNA break site [54, 55]. Targeted treatment to JNK and PARP1 was validated to overcome chemoresistance by suppressing chemotherapeutic drug-induced DSB repair.

DSB is most dangerous for chemotherapeutic drug-induced DNA damage. Intrinsic DSB repair capacity determines tumor cell fate. We demonstrated that miRNA-363-3p-related chemoresistance can be attributed to enhanced DSB repair function in DLBCL. Interestingly, miRNA-363-3p acted via NHEJ (including alternative NHEJ) rather than HR. Actually, the activity of HR repair was decreased by miRNA-363-3p. HR is an error-free DSB repair route and relies on the sister chromatid to provide a precisely matched repair template. Gene sequencing revealed the association of gene mutation in HR repair pathway with HR deficiency, such as *BRCA1* and *BRCA2* mutation. Loss of HR repair usually makes tumor cells more addiction to error-prone rejoining repair, i.e., alternative NHEJ [56]. PARP1 inhibitors have been approved to target the HR-deficient phenotype of *BRCA1*- or *BRCA2*-mutant tumors as a paradigm for synthetic lethality strategies [56, 57]. As we know, *BRCA1* and *BRCA2* mutations are rare in DLBCL, and HR deficiency in DLBCL was much less addressed. In this study, we discovered a novel mechanism of HR suppression regulated by miRNA-363-3p, and PARP1 inhibition significantly increased the anti-tumor activity of chemotherapeutic drugs in this context.

In summary, we creatively put forward a novel anthracycline-based chemoresistance mechanism involving miRNA-363-3p/DUSP10/JNK-mediated enhancement of DSB repair. New approaches targeting this resistance mechanism provide potential options for overcoming chemoresistance.

## Supplementary information

Supplmentary Figure and table

## Data Availability

The materials are described in the manuscript and supplementary information is posted on the journal’s website. For other original data, please contact yyliu@zzu.edu.cn.
