# Supplementary material for: MiRNA-363-3p/DUSP10/JNK axis mediates chemoresistance by enhancing DNA damage repair in diffuse large B-cell lymphoma
Source: Leukemia. 2022 Apr 29;36(7):1861–9. doi: 10.1038/s41375-022-01565-6 (PMC9252898; doi:10.1038/s41375-022-01565-6)
Supplement: Supplementary file 1 — Supplmentary Figure and table [file 41375_2022_1565_MOESM1_ESM.pdf]

Supplmentary Figure 1

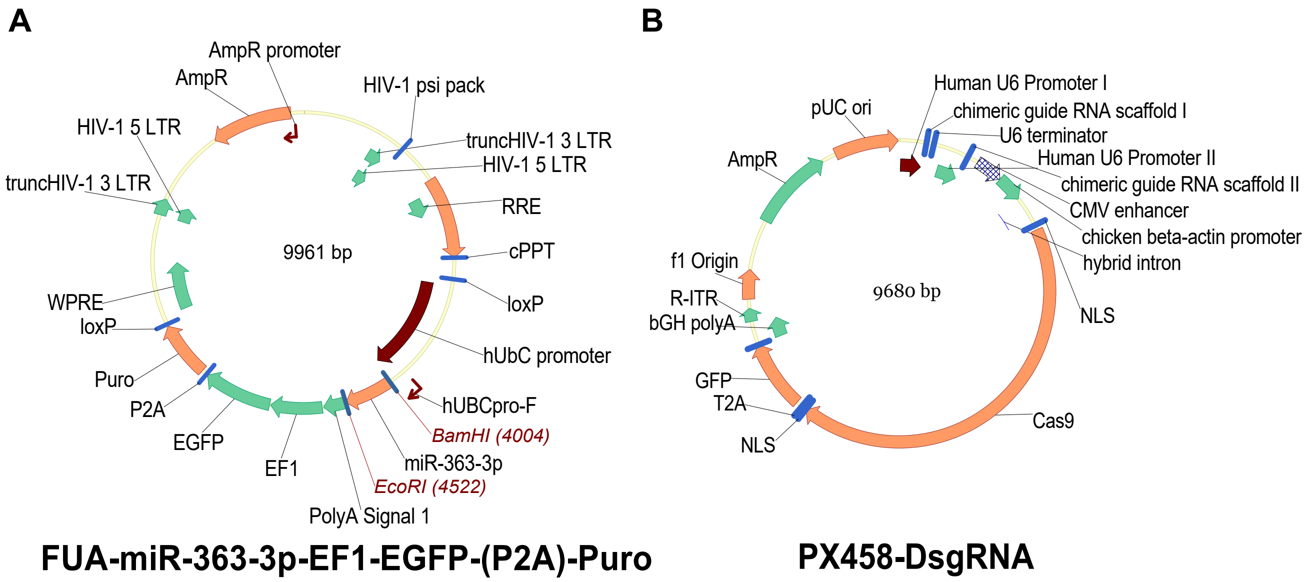

Supplementary Figure 1: Lentivirus vector for miR-363-3p-ectopic expression and PX458 derived dual sgRNA vector for miR-363-3p knockout.

Supplementary Figure 2

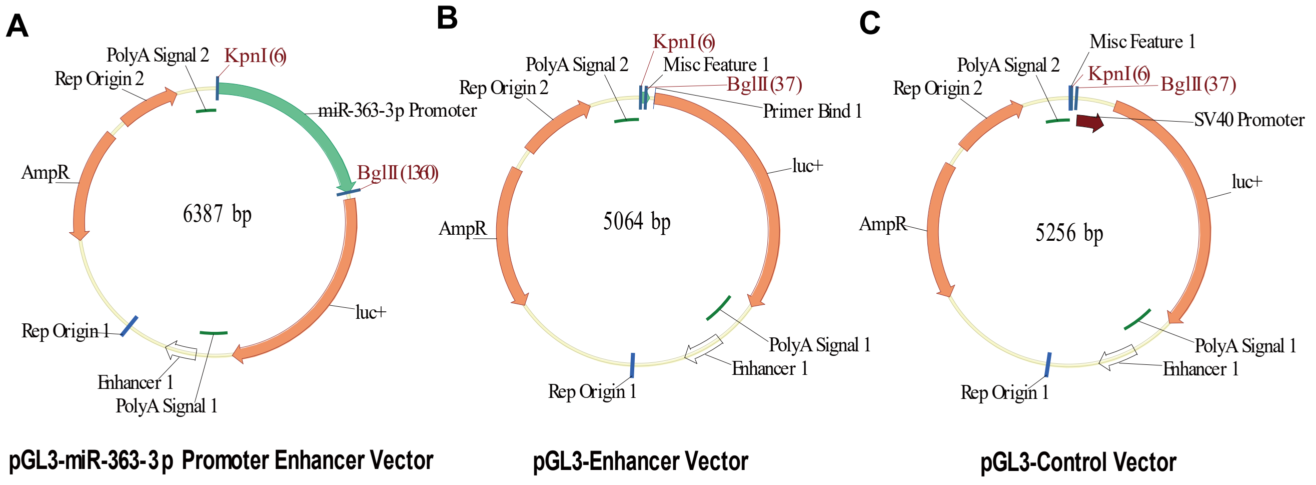

Supplementary Figure 2: Luciferase report system for validating the function of miR-363-3p promoter.

Supplmentary Figure 3

A

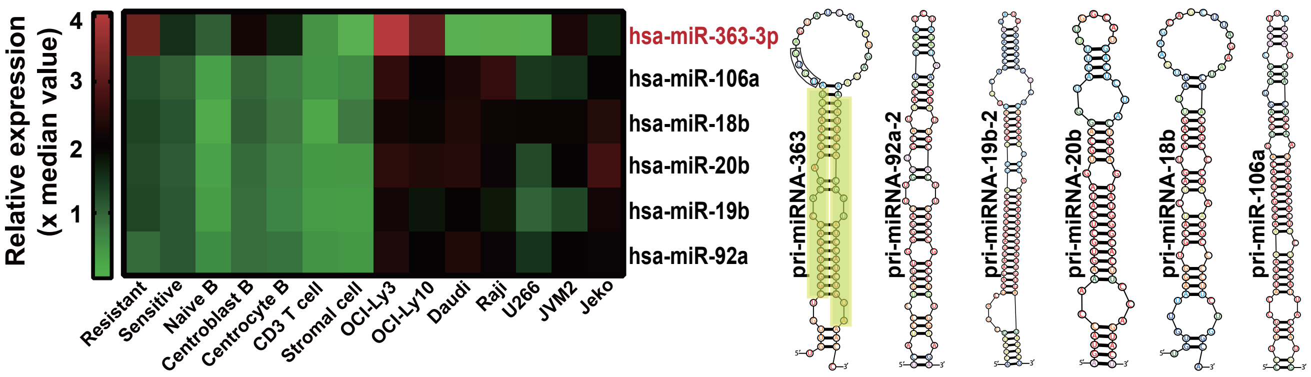

Supplementary Figure 3 A: Each member of the miRNA-106a-363 cluster is illustrated using gene heat map in different contexts. An obvious difference was observed among the secondary structure of precursor miRNA moiety.

B

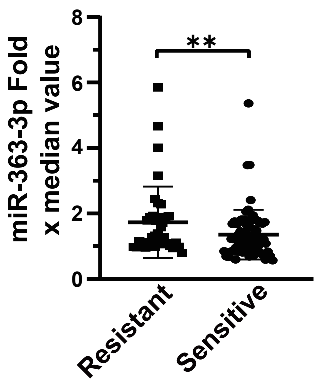

D

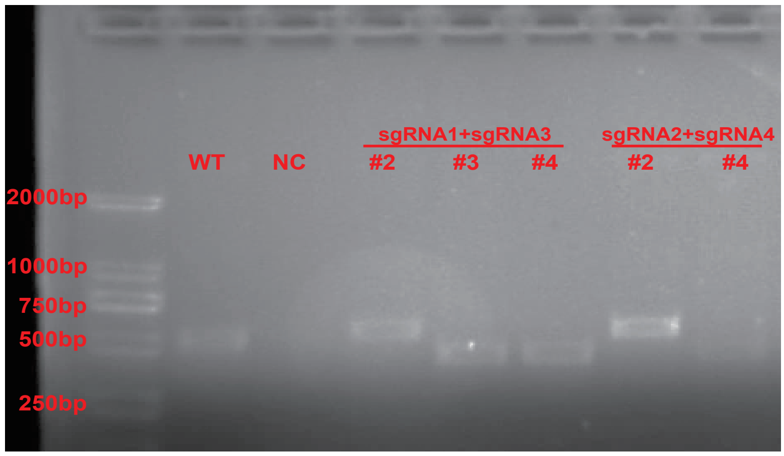

Supplementary Figure 3 B: Quantitative RT PCR analysis showed that the level of miRNA-363-3p was higher in resistant (n = 37) than sensitive patients (n = 69, ***p* < 0.01) in a larger DLBCL cohort (n = 106).

Supplementary Figure 3 D: single clone electropherograms of the miR-363-3p knockout in OCI-Ly3.
WT: wild-type genomic DNA;NC: no-template control;
sgRNA1+sgRNA3(sg1+3 in main text): Dual sgRNA-directed miR-363-3p knockout by CRISPR/Cas9 with sgRNA1 and sgRNA3;
#: number sign of single clone.

C

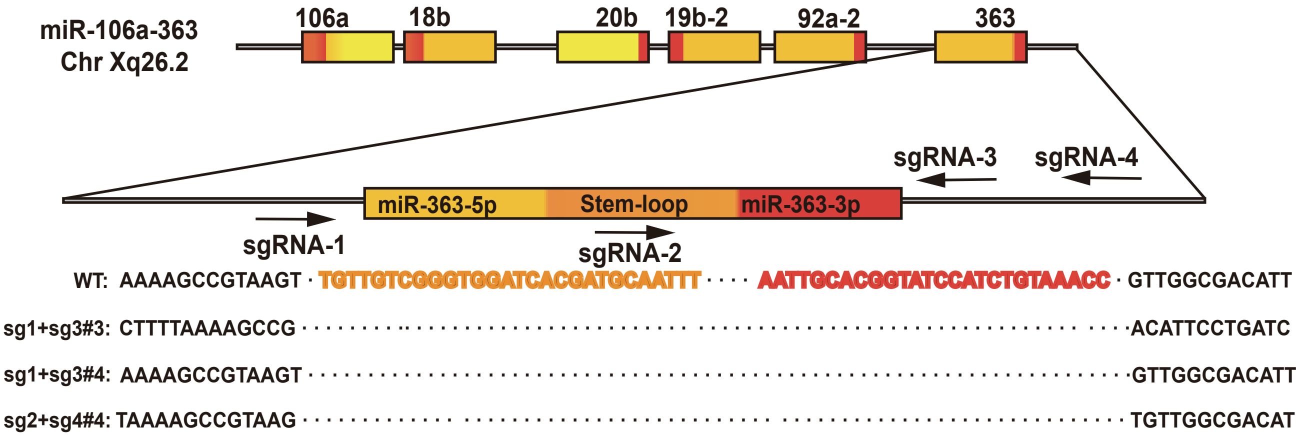

Supplementary Figure 3 C: Scheme of Cas9/sgRNA-targeting sites (black arrows) in miR-363 genome locus (yellow: miR-363-5p; orange: Stem-loop; red: miR-363-3p).
WT: wild-type genomic DNA;
sgRNA1+sgRNA3(sg1+3 in main text): Dual sgRNA-directed miR-363-3p knockout by CRISPR/Cas9 with sgRNA1 and sgRNA3;
#: number sign of single clone.

Supplmentary Figure 4

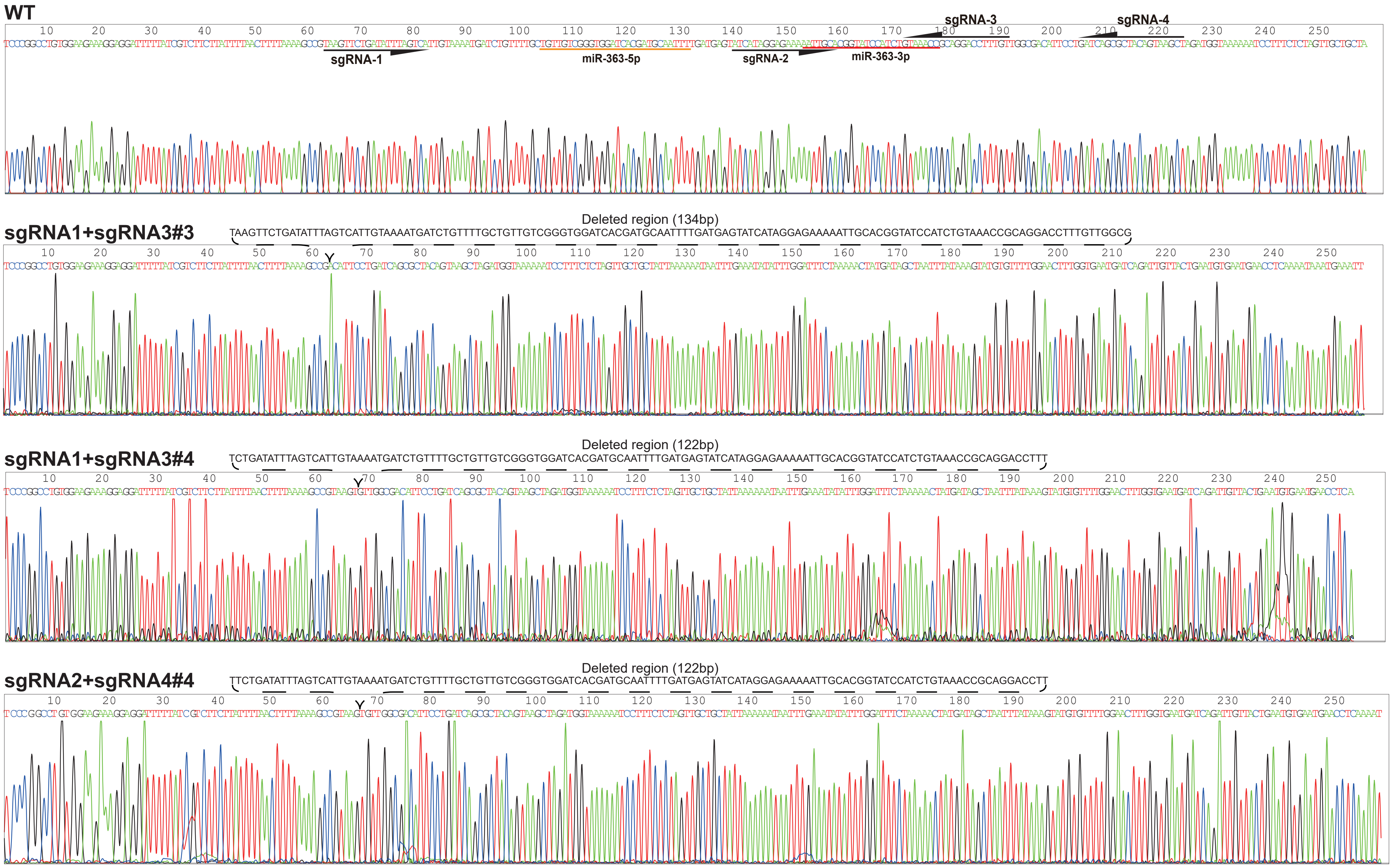

Supplementary Figure 4: The sanger sequencing of single clone with miR-363-3p knockout in OCI-Ly3.

WT: wild-type genomic DNA;

sgRNA1+sgRNA3(sg1+3 in main text):Dual sgRNA-directed miR-363-3p knockout by CRISPR/Cas9 with sgRNA1 and sgRNA3;

#: number sign of single clone.

Supplmentary Figure 5

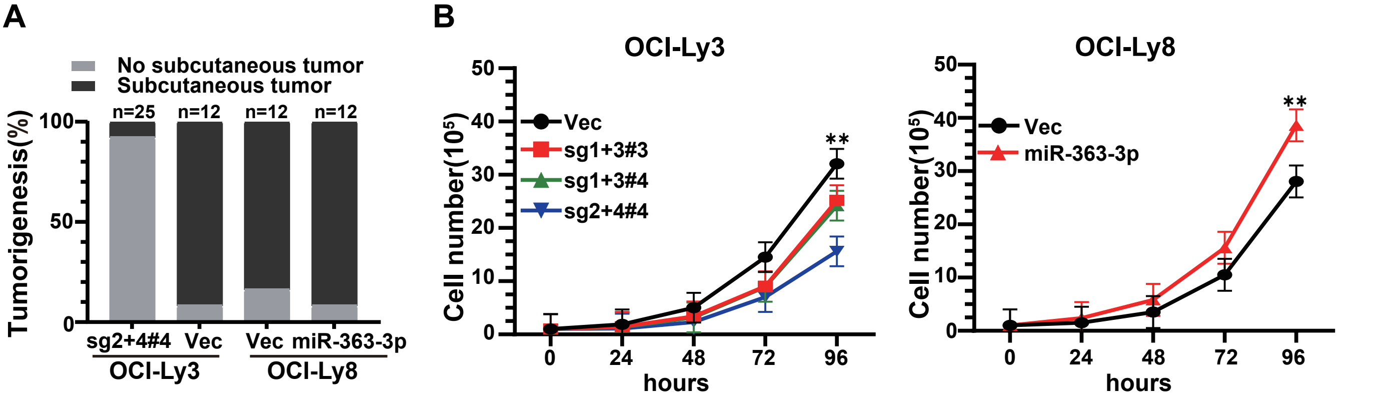

Supplementary Figure 5 A: miRNA-363-3p-knockout OCI-Ly3 cells (sg2+sg4#4) produced the lowest efficiency of tumorigenesis.

sg2+4: Dual sgRNA-directed miR-363-3p knockout by CRISPR/Cas9 with sgRNA2 and sgRNA4;
#: number sign of single clone.

Supplementary Figure 5 B: Cell viability assay indicated the obvious increase in miRNA-363-3p-ectopic OCI-Ly8 and decrease in miRNA-363-3p-knockout OCI-Ly3 (**$p < 0.01$).

sg1+3: Dual sgRNA-directed miR-363-3p knockout by CRISPR/Cas9 with sgRNA1 and sgRNA3;
#: number sign of single clone.

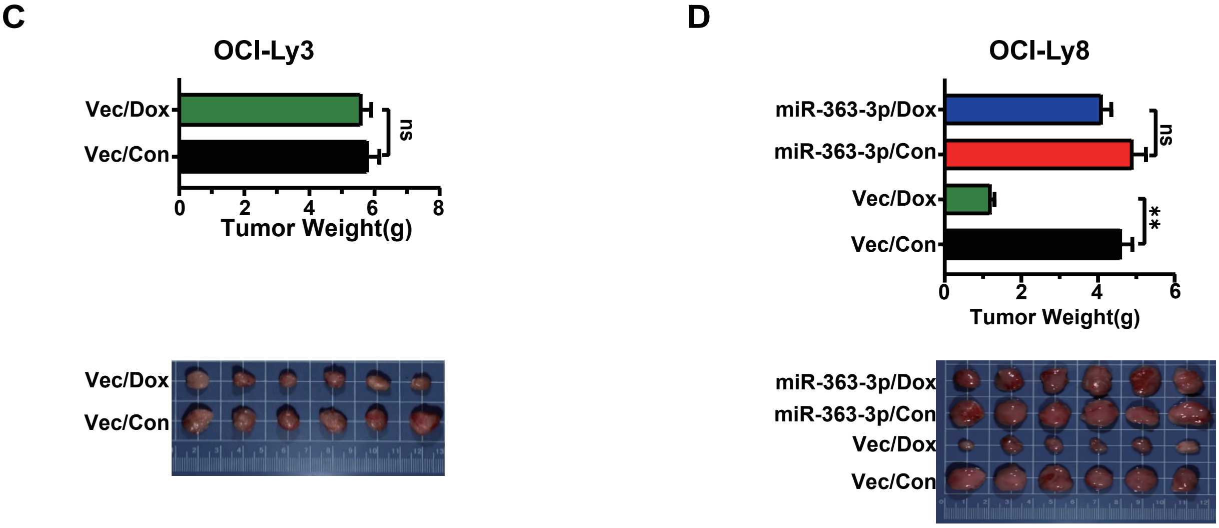

Supplementary Figure 5 C: Doxorubicin induced a modest tumor suppression in OCI-Ly3 vector-bearing mice (n = 6 each).

Supplementary Figure 5 D: Doxorubicin induced significant tumor suppression in OCI-Ly8 vector control-bearing mice (n = 6 each), which was significantly decreased in miRNA-363-3p-ectopic mice (n = 6 each) (**$p < 0.01$).

Supplmentary Figure 6

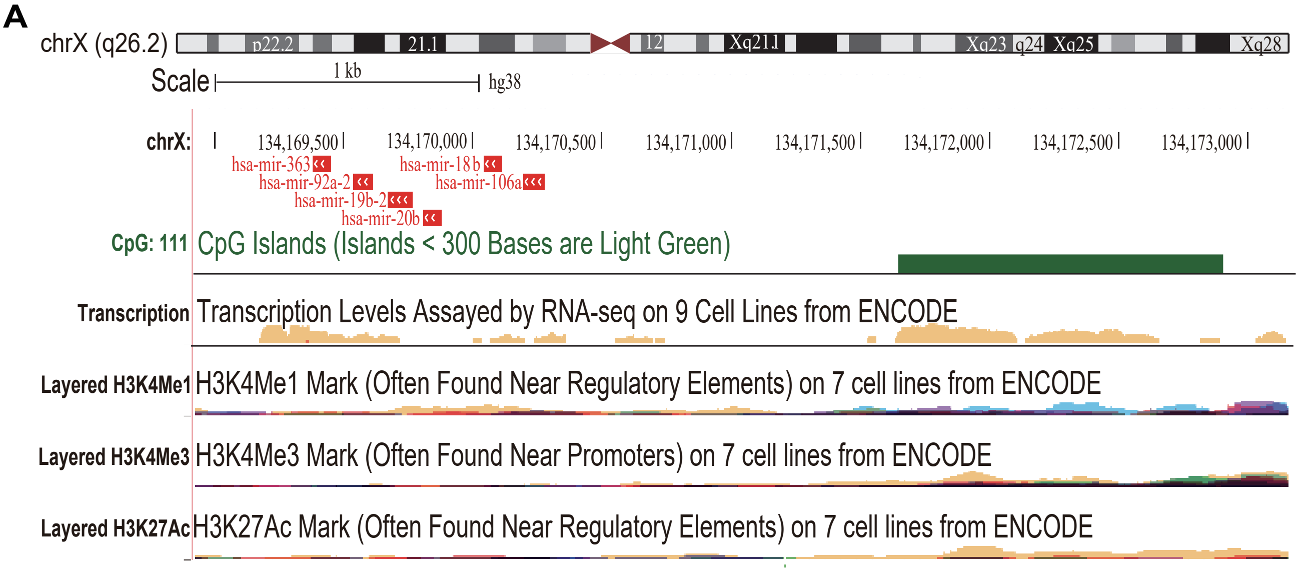

Supplementary Figure 6 A: Schematic representation of miR-363 genomic locus. The miR-363 location (red box), CpG island position (green box), and transcriptionally active promoter maker H3K4me3, H3K27Ac (wave shape) were indicated.

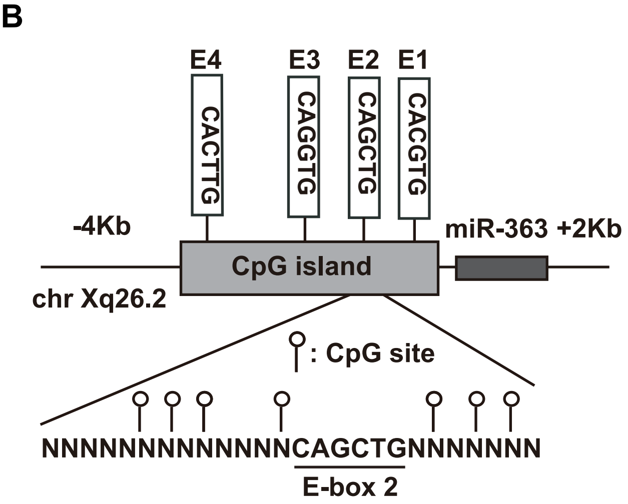

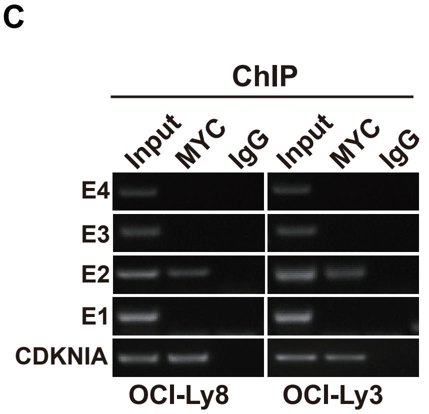

Supplementary Figure 6 B: The scheme of miRNA-363-3p promoter showed four E-boxes in CpG island.

Supplementary Figure 6 C: Chromatin immunoprecipitation (ChIP) combining with PCR assay revealed precise binding of MYC to E-box-2 site and higher binding efficiency in OCI-Ly3 than in OCI-Ly8 cells.

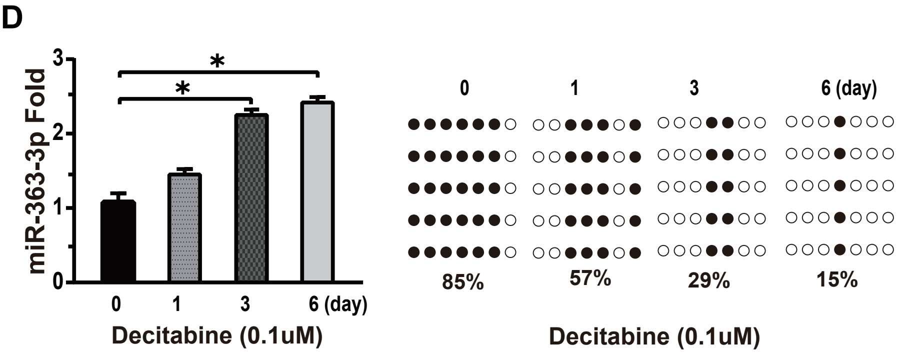

Supplementary Figure 6 D: Quantitative RT PCR analysis found the significant increase of miRNA-363-3p level in OCI-Ly8 cells after decitabine (0.1 μM) treatment for 1, 3 and 6 days(*$p < 0.05$). BSP analysis indicated the obvious reduction of methylation alleles surrounding E-box-2 in OCI-Ly8 cells after decitabine (0.1 μM) treatment for 1, 3 and 6 days.

●: Methylation, ○: Unmethylation.

Each row represents a single cloned allele.

Supplmentary Figure 7

A

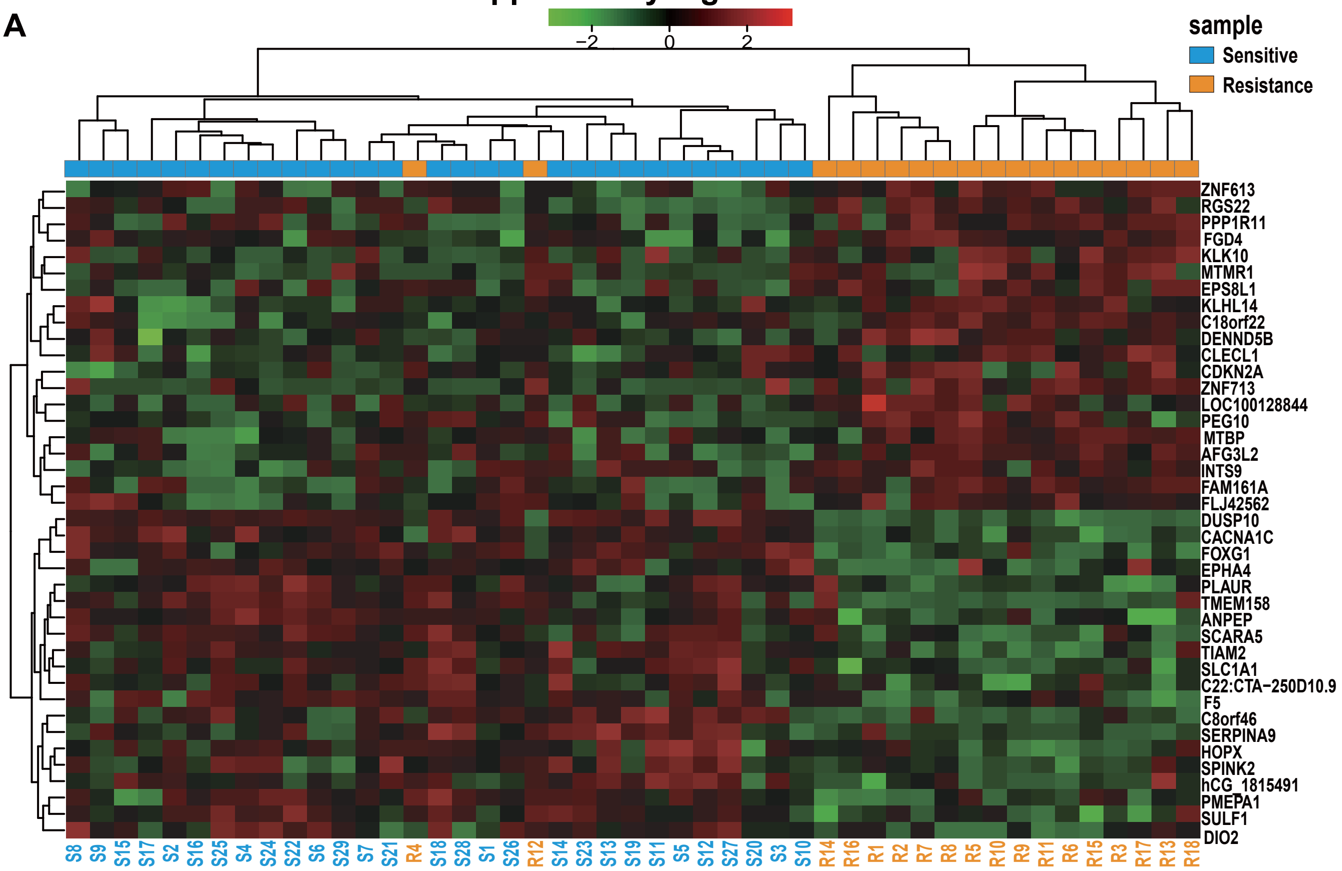

Supplementary Figure 7 A: Unsupervised hierarchical clustering of GEP data from 47 DLBCL patients revealed many genes in association with R-CHOP resistance. The heat map represents top 20 differential genes enriched in resistant (R) and sensitive (S) group.

B

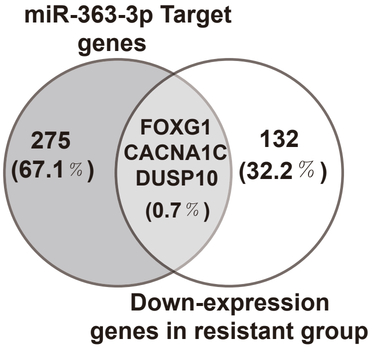

Supplmentary Figure 7 B: Venn diagram showed the overlapping between miRNA-363-3p targets and down-expression genes in R group, including FOXG1, CACNA1C, and DUSP10.

D

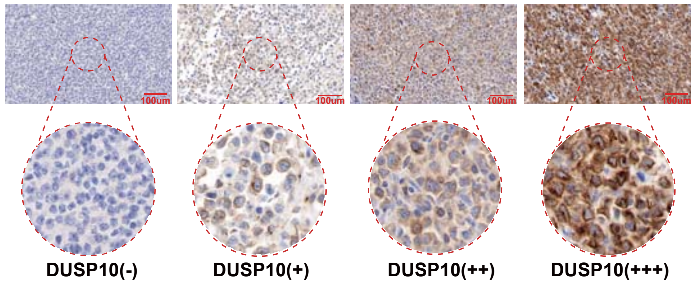

E

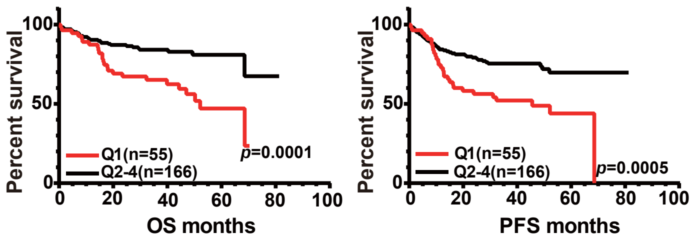

Supplmentary Figure 7 D: Representing Images of immunohistochemical staining for DUSP10 expression validated in 106 DLBCL patients (400X).

Supplmentary Figure 7 E: Kaplan-Meier and Log-Rank analyses showed the significant association of low DUSP10 expression (Q1) with unfavorable OS (p = 0.0001) and PFS (p = 0.0005) in another larger DLBCL cohort (n = 221).

C

DUSP10 3´UTR 5´••••••••GCCUUCACUGGUUCUU<u>GUGCAAU</u>AAUAUG••••••••3´

miR-363-3p 3´••••••••AUGUCUACCUAUGG<u>CACGUUA</u>A••••••••5´

FOXG1 3´UTR 5´••••••••GUGGAGUGUCAGCGAG<u>GUGCAAU</u>GUGGGG••••••••3´

miR-363-3p 3´••••••••AUGUCUACCUAUGG<u>CACGUUA</u>A••••••••5´

Supplmentary Figure 7 C: Bioinformatics analysis (TargetScan) of the predicted miR-363-3p binding site in the 3′UTR of the DUSP10 and FOXG1 gene.

Supplmentary Figure 8

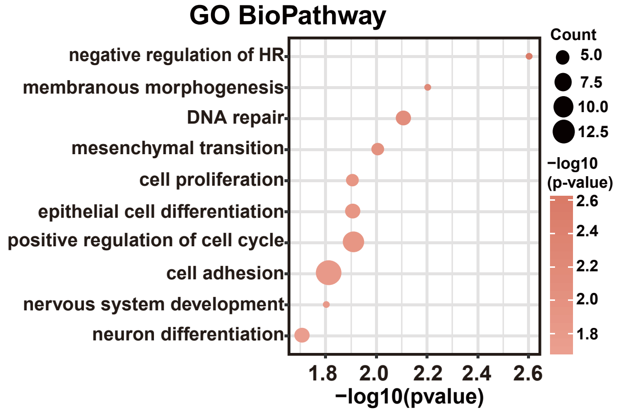

Supplementary Figure 8 A: David pathway analysis revealed the enrichment of biological pathways relevant to R-CHOP resistance.

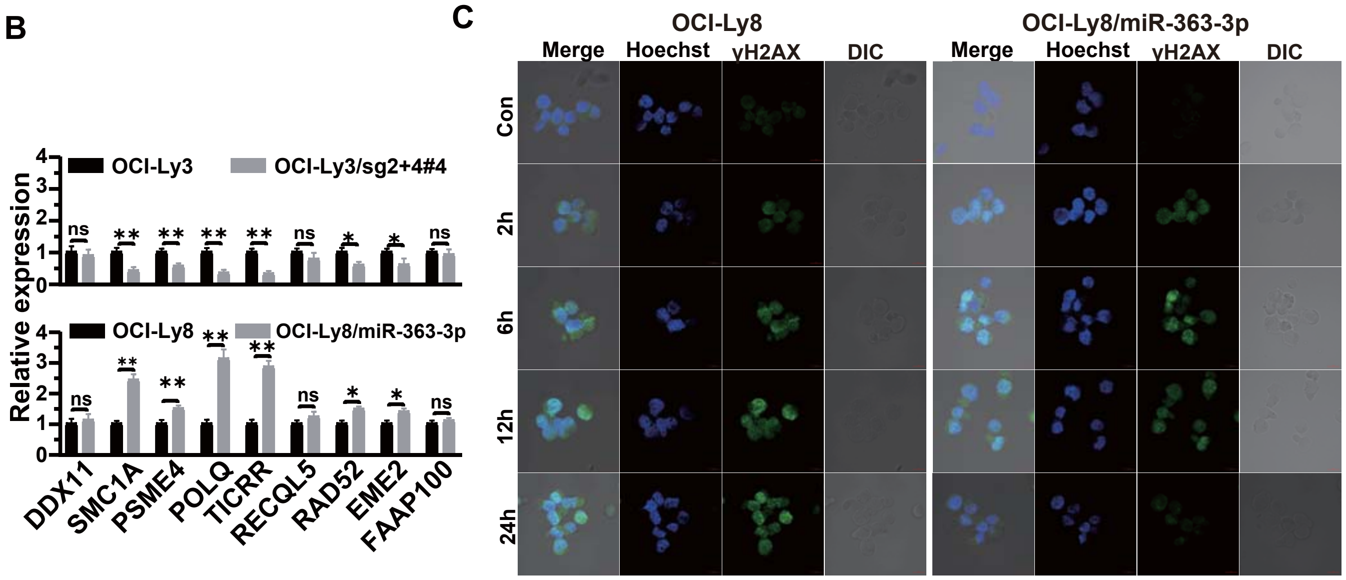

Supplementary Figure 8 B: Quantitative RT PCR analysis showed that the level of nine genes was positively associated with the miRNA-363-3p level(**p* < 0.05, ***p* < 0.01).

Supplementary Figure 8 C: Immunofluorescence assays showed that the level of doxorubicin (25ng/ml) -induced γH2AX was gradually reduced in miRNA-363-3p-ectopic OCI-Ly8 cells and increased in miRNA-363-3p-knockout OCI-Ly3 cells after doxorubicin withdrawal of 0, 2, 6, 12, and 24 hours.

Supplmentary Figure 9

A

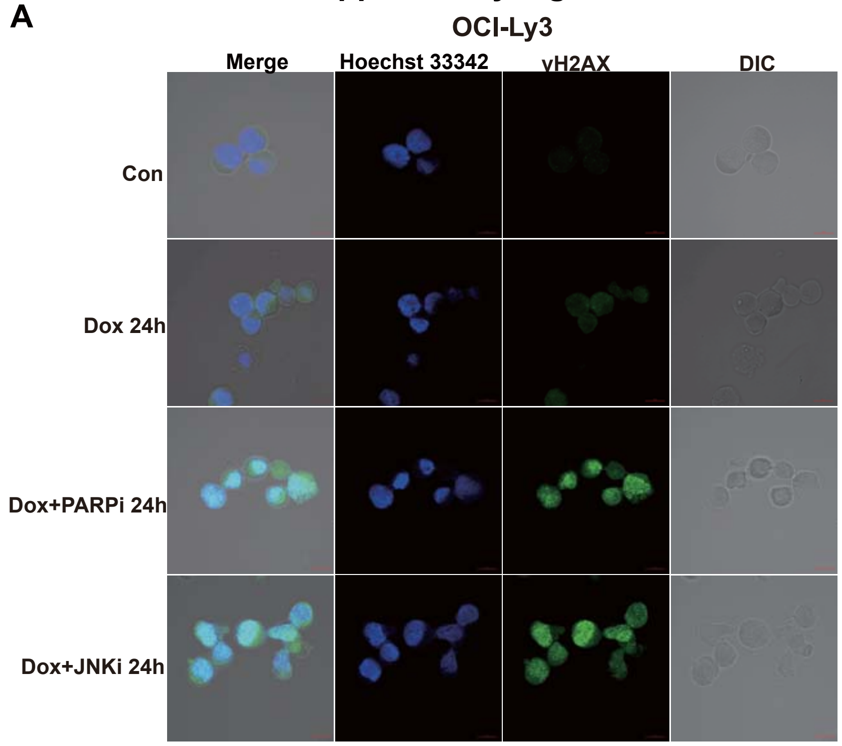

Supplementary Figure 9 A: Immunofluorescence assays showed that SP600125 (20 μM, JNK1/2/3 inhibitor) and BGB-290 (25 μM, PARP1 inhibitor) obviously increased doxorubicin (25 ng/ml)-induced γH2AX level in OCI-Ly3 cells after treatment for 0, 2, 6, 12, and 24 hours.

B

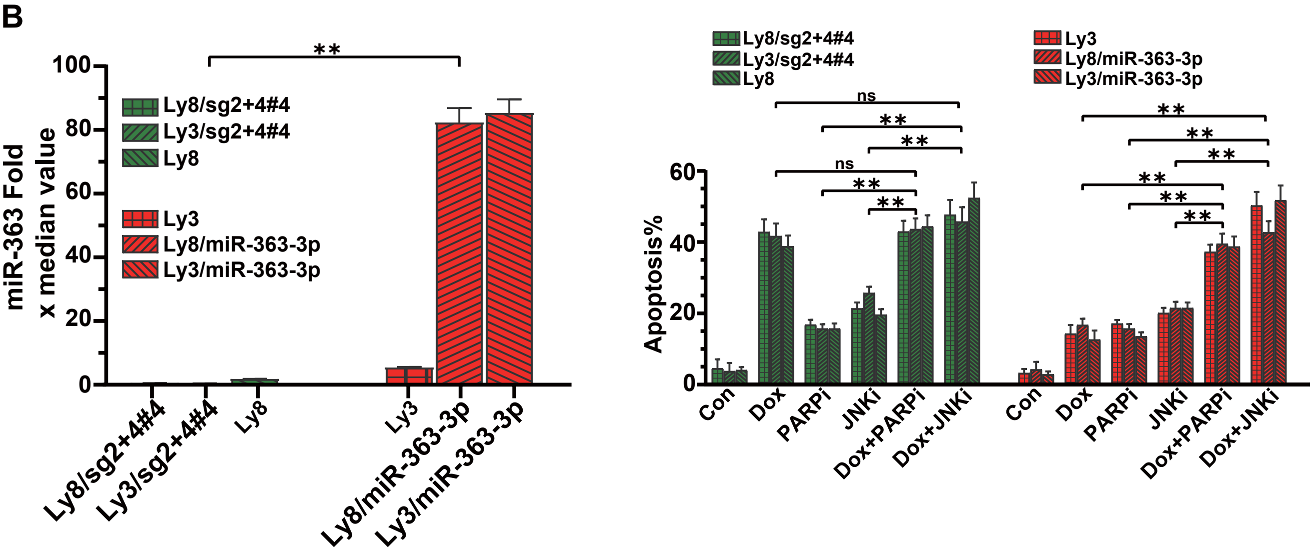

Supplmentary Figure 9 B: miR-363-3p expression levels in DLBCL cells. Flow cytometry analysis revealed that SP600125 (20 μM, JNK1/2/3 inhibitor) and BGB-290 (25 μM, PARP1 inhibitor) obviously promoted doxorubicin (25 ng/ml)-induced apoptosis in DLBCL cell lines with high expression of miRNA-363-3p after treatment for 48 hours.The histograms present the values from at least 3 experiments (***p* < 0.01).

C

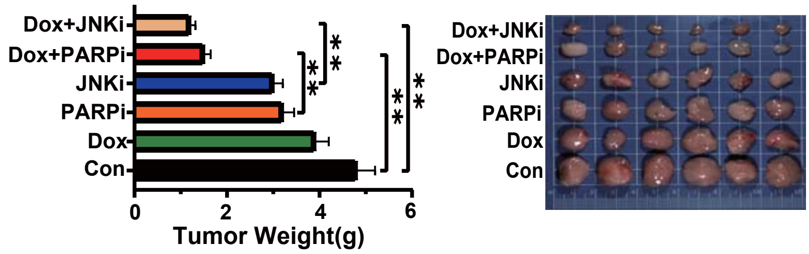

Supplmentary Figure 9 C: SP600125 (25 mg/kg, JNK1/2/3 inhibitor) and BGB-290 (25 mg/kg, PARP1inhibitor) significantly enhanced doxorubicin (20 mg/kg)-induced tumor suppression in mice bearing OCI-Ly3 cells (n = 6 each) (***p* < 0.01).

Supplementary Table 1:
Comparison of clinicopathological features between resistant and sensitive groups

Characteristics	Resistant group (n=37)	Sensitive groups (n=69)	*p* value	
			univariant	multivariant
**Gender**				
Male, n (%)	24 (64.9)	33 (47.8)	0.095	0.293
**Age (ys)**				
Median, (range)	56 (32-78)	58 (19-76)	0.725	0.805
**Performance status**				
ECOG score > 1, n (%)	5 (13.5)	8 (11.6)	0.852	0.804
**LDH level**				
High, n (%)	21 (56.8)	22 (31.9)	0.009	0.014
**Ann Arbor stage**				
Stage III or IV, n %)	24 (64.9)	31 (44.9)	0.051	0.097
**Extranodal involvement**				
The number >1 , n(%)	7 (18.9)	10 (14.5)	0.558	0.428
**IPI**				
Score 3-5, n (%)	9 (24.3)	12 (17.4)	0.278	0.210
**Cell of origin**				
Non-germinal center, n (%)	11 (29.7)	27 (39.1)	0.377	0.464
**MYC status**				
Positive, n (%)	10 (27.0)	17 (24.6)	0.822	0.748
**BCL2 status**				
Positive, n (%)	29 (78.4)	47 (68.1)	0.352	0.170
**TP53 status**				
Positive, n (%)	7 (18.9)	14 (20.3)	0.812	0.863
**miRNA-363-3p level (x median value)**				
Median (range)	2.452 (0.562-6.741)	0.621 (0.078-8.136)	0.0085	0.031

Supplementary Table 2
list of the primer sequences

**Clone miRNA-363 into lentiviral vector**	Forward Primer	5'-AACTGATTGTGATAATGTGTGCTTCC-3'
	Reverse Primer	5'-TTTTGAGGTTCATTCACATTCAGTAA-3'
**Primer for methylation analysis**	Forward primer	5'-AGGGGAGTTTTTTTTTTTTTTT-3'
	Outside Reverse primer	5'-TTAATAAACRCTTTTTCTCACTTCT-3'
	Inside Reverse primer	5'-CTCACTTCTCTTCCCCTTCTAACT-3'
**sgRNA for miRNA-363 knockout**	sgRNA-1	5'-TGACTAAATATCAGAACTTA-3'
	sgRNA-2	5'-ATCATAGGAGAAAAATTGCA-3'
	sgRNA-3	5'-GTAAACCGCAGGACCTTTGT-3'
	sgRNA-4	5'-AGCTTACTGTAGCGCTGATC-3'
**Clone the promoter of miRNA-363**	Forward Primer	5'-ATCggtaccCGTGTTTCTATTTCTTCCCTCCTC-3'
	Reverse Primer	5'-ATCagatctAAGTCCTTCCCTGTACGCCTTTGT-3'
**Primer for ChIP-PCR**	E1-Forward Primer	5'-CCTCCATTCCCGGGTTGGTG-3'
	E1-Reverse Primer	5'-CTCCACCGTTGACCTTTAGG-3'
	E2-Forward Primer	5'-CCGCTACCCCTCAACCGCACT-3'
	E2-Reverse Primer	5'-GGTGGCTGGGCAAGAAGGGAG-3'
	E3-Forward Primer	5'-CTCCCTTCTTGCCCAGCCACC-3'
	E3-Reverse Primer	5'-TGGAGGGGACACCCGGAGA-3'
	E4-Forward Primer	5'-AAGCGTCCACTAAATGGATCA-3'
	E4-Reverse Primer	5'-TTGGGCTATTAAAGGCAGAG-3'
**Primer for RT-PCR**	DDX11-Forward Primer	5'-TGGAACTGGCCCCTTACATGA-3'
	DDX11-Reverse Primer	5'-CTGCACAAACTGAGTAACCCA-3'
	SMC1A-Forward Primer	5'-CTGGCGCAGGAGTATGACAAG-3'
	SMC1A-Reverse Primer	5'-CCCGTACTACCTCATCCTTCA-3'
	PSME4-Forward Primer	5'-TGGGAAGCAGTCAAGTGTTAGT-3'
	PSME4-Reverse Primer	5'-CAGCGCCCATTATTTGAAGGA-3'
	POLQ-Forward Primer	5'-CTGCGTCGGAGTGGGAAAC-3'
	POLQ-Reverse Primer	5'-CTGTAGGCTTGCATTCTCCTG-3'
	TICRR-Forward Primer	5'-CCTCACCTATCTGAGTTGCCG-3'
	TICRR-Reverse Primer	5'-CACTGGTAGTCTAGCAGCGT-3'
	RECQL5-Forward Primer	5'-AAGAAACCAGTTGCCATCTTCA -3'
	RECQL5-Reverse Primer	5'-TCAGGTTCCCATAGGGATCAG-3'
	RAD52-Forward Primer	5'-CCAGAAGGTGTGCTACATTGAG-3'
	RAD52-Reverse Primer	5'-ACAGACTCCCACGTAGAACTTG-3'
	EME2-Forward Primer	5'-CGCCGTTACCAAGGCTCTC-3'
	EME2-Reverse Primer	5'-GCTGACCCGACTGAACTGC-3'
	FAAP100-Forward Primer	5'-CTCCTGTGCTCTCGACCTG-3'
	FAAP100-Reverse Primer	5'-CTGAGACTGTAGAACAGCGTG-3'
**DUSP10 and FOXG1 WT and Mut 3' UTRs**	DUSP10-WT-Forward	5'-CTGACCATTATATGCCTTCACTGGCTTCTT**GTGCAATA**ATATGATGTTTTAAGTGTGCAA-3'
	DUSP10-WT-Reverse	5'-TTGCACACTTAAAACATCATAT**TATTGCAC**AAGAAGCCAGTGAAGGCATATAATGGTCAG-3'
	DUSP10-Mut-Forward	5'-CTGACCATTATATGCCTTCACTGGCTTCTT***AGTTGGCG*** ATATGATGTTTT AAGTGTGCAA-3'
	DUSP10-Mut-Reverse	5'-TTGCACACTTAAAACATCATAT***CGCCAACT*** AAGAAGCCAGTGAAGGCATATAATGGTCAG-3'
	FOXG1-WT-Forward	5'-AACAAGGTGTGGAGTGTCAGCGAG**GTGCAAT**GTGGGGAGAATACATTGTAGAATATAAGG-3'
	FOXG1-WT-Reverse	5'-CCTTATATTCTACAATGTATTCTCCCCAC**ATTGCAC**CTCGCTGACACTCCACACCTTGTT-3'
	FOXG1-Mut-Forward	5'-AACAAGGTGTGGAGTGTCAGCGAG***AGTTGGC*** GTGGGGAGAATACATTGTAGAATATAAGG-3'
	FOXG1-Mut-Reverse	5'-CCTTATATTCTACAATGTATTCTCCCCAC***GCCAACT*** CTCGCTGACACTCCACACCTTGTT-3'

Amplification condition for methylation analysis

**First Round: Forward primer, Outside Reverse primer**	94℃	60℃(-1℃ per cycle,10 cycles)	72℃
	30s	1min	1min
	94℃	51℃(45 cycles)	72℃
	30s	1min	1min
**Second Round: Forward primer, Inside Reverse primer**	94℃	55℃(35 cycles)	72℃
	30s	1min	1min

Supplementary Table 3
list of the Antibody

	Antibody	Company	Cat No	Working	molecular weight	Application
**primary antibody**	Anti-Histone H3 antibody	Abcam	ab1791	1:2000	17 kDa	WB
	Anti-DUSP10 antibody	Abcam	ab140123	1:1000	53 kDa	WB
	Anti-gamma H2A.X (phospho S139)	Abcam	ab11174	1:2000	15 kDa	WB
	Phospho-Histone H2A.X (Ser139)	CST	#9718	1:400	15 kDa	IF
	SAPK/JNK Antibody	CST	#9252	1:1000	46 kDa, 54 kDa	WB
	Phospho-SAPK/JNK (Thr183/Tyr185) (81E11) Rabbit mAb	CST	#4668	1:1000	46 kDa, 54 kDa	WB
	p38 MAPK Antibody	CST	#9212	1:1000	40kDa	WB
	Phospho-p38 MAPK (Thr180/Tyr182) (D3F9) XP® Rabbit mAb	CST	#4511	1:1000	43kDa	WB
	Anti-p53 antibody	Abcam	ab26	1:1000/1:500	53 kDa	WB/IHC
	Anti-p53 (phospho S15) antibody	Abcam	ab1431	1:1000	53 kDa	WB
	Anti-Bcl-2 antibody	Abcam	ab182858	1:2000/1:500	26 kDa	WB/IHC
	ATF-2 Antibody	Santa Cruz	sc-187	1:1000	70kDa	WB
	Anti-p-ATF-2 Antibody	Santa Cruz	sc-8398	1:1000	70kDa	WB
	Anti-MKP-5 Antibody	Santa Cruz	sc-374276	1:100	53 kDa	IHC
	Anti-Myc/c-Myc Antibody	Santa Cruz	sc-40	1:1000/1:200	67kDa	WB/IHC
	Anti-Myc/c-Myc Antibody (9E10) B	Santa Cruz	sc-40 B	200ug/ml	67kDa	CHIP
	Recombinant Anti-CD10 antibody	Abcam	ab256494	1:500	85 kDa	IHC
	Recombinant Anti-Bcl6 antibody	Abcam	ab172610	1:250	78 kDa	IHC
	Recombinant Anti-MUM1 antibody	Abcam	ab133590	1:250	52 kDa	IHC
**secondary antibody**	Goat anti-Rabbit IgG (H+L)-Alexa Fluor 488	Invitrogen	#A-11008	1:500		IF
	Goat Anti-Rabbit IgG (H+L)-HRP	Abcam	ab205718	1:2000/1:20000		WB/IHC
	Rabbit Anti-Mouse IgG (H+L)-HRP	Abcam	ab6728	1:2000/1:200		WB/IHC
